# Effects of exercise interventions on depressive symptoms in perimenopausal women in China: a systematic review and meta-analysis

**DOI:** 10.3389/fpsyg.2026.1848514

**Published:** 2026-07-22

**Authors:** Shuhan Dong, Tina Chan, Yuhong Wen

**Affiliations:** 1 School of Recreational Sports and Tourism, Beijing Sport University, Beijing, China; 2 School of Social Science, Tsinghua University, Beijing, China

**Keywords:** depression, exercise, meta-analysis, perimenopause, physical activity, women’s mental health

## Abstract

**Objective:**

Perimenopausal women are at increased risk of depressive symptoms due to hormonal and psychosocial changes, while evidence regarding the effectiveness of exercise interventions in Chinese populations remains limited and inconsistent. This study aimed to systematically evaluate the effects of exercise interventions on depressive symptoms in Chinese perimenopausal women and to provide evidence for non-pharmacological intervention strategies.

**Methods:**

This systematic review and meta-analysis was reported in accordance with the PRISMA guidelines and conducted based on the PICOS framework. Web of Science, PubMed, Cochrane Library, CNKI, Wanfang, and VIP databases were searched from inception to December 2025. Randomized controlled trials investigating exercise interventions in Chinese perimenopausal women were included. Two reviewers independently screened studies, extracted data, and assessed risk of bias using the Cochrane risk-of-bias tool. Meta-analysis was conducted using Stata 16.0 with standardized mean differences (SMDs) and 95% confidence intervals (CIs). Random-effects models, subgroup analyses, sensitivity analyses were performed to explore heterogeneity.

**Results:**

A total of 19 studies comprising 21 randomized controlled trials involving 1,398 Chinese perimenopausal women were included. Compared with control conditions, exercise interventions significantly reduced depressive symptoms (SMD = −1.37, 95% CI: −2.06 to −0.69, *p* < 0.001), although substantial heterogeneity was observed (I^2^ = 97.14%). Sensitivity analyses indicated that the pooled results were relatively stable after sequential exclusion of individual studies. Begg’s and Egger’s tests suggested potential publication bias (*p* < 0.05); however, trim-and-fill analysis showed that the overall pooled effect remained statistically significant. Subgroup analyses suggested that exercise interventions with different session durations, frequencies, intensities, and intervention periods were associated with improvements in depressive symptoms, although substantial heterogeneity remained across subgroups.

**Conclusion:**

Exercise interventions may effectively alleviate depressive symptoms in Chinese perimenopausal women and represent a feasible non-pharmacological strategy for mental health management. However, given the substantial heterogeneity and potential publication bias among included studies, the findings should be interpreted cautiously. Further high-quality randomized controlled trials with standardized intervention protocols and long-term follow-up are needed to determine the optimal exercise prescription for this population.

**Systematic review registration:**

https://www.crd.york.ac.uk/PROSPERO/view/CRD420251271853, identifier CRD420251271853.

## 1 Introduction

With the accelerating process of global population aging, the physical and mental health of perimenopausal women has received increasing attention. According to the prediction of the World Health Organisation (WHO), the global number of perimenopausal women will exceed 1.2 billion by 2030 (Li, 2018), among which the population in China will surpass 210 million (Huang et al., 2025). WHO defines perimenopause as the period from the onset of ovarian function decline to 1 year after menopause (Lu and Huang, 2025). During this period, the decline in ovarian function and the sharp drop in estrogen levels may induce a series of physiological and psychological symptoms (Zhou, 2024), such as irritability, joint pain, memory impairment and depression (Weber et al., 2021). Among these, depressive symptoms have become an important public health issue affecting the quality of life and social functioning of perimenopausal women (Zhang et al., 2026). Specifically, the incidence of depression among this population ranges from 45 to 68% (Maki et al., 2019). Furthermore, among perimenopausal women, the more severe the amenorrhea symptoms and the higher the level of somatic stress, the higher the risk of depression (Lee and Kim, 2010).

In recent years, exercise intervention, as a safe, economical, and sustainable non-pharmacological treatment, has gradually become a research focus in the field of mental health intervention for perimenopausal women (Liu et al., 2025). Evidence suggests that regular exercise is associated with improvements in women’s physical function, physiological health, mental well-being, and overall health status (Wang et al., 2021). Previous studies have consistently reported that various forms of exercise, including walking, aerobic exercise, and traditional Chinese exercises, can alleviate depressive symptoms and improve related psychological outcomes such as anxiety and sleep disturbances among perimenopausal women (Abedi et al., 2015; Sternfeld et al., 2014; Chang et al., 2011; Ding, 2025). These findings suggest that exercise may serve as an effective non-pharmacological strategy for improving mental health during the perimenopausal period.

Although numerous studies have confirmed that exercise intervention exerts a certain improving effect on depression in perimenopausal women (Cramer et al., 2018; Pereira et al., 2012), there are still some deficiencies and controversies in the existing studies: first, most studies have a small sample size, resulting in limited stability and representativeness of the research results (Wang et al., 2017; Sun et al., 2023); second, there are significant differences in exercise intensity, cycle and frequency adopted in different studies, with a lack of systematic classification and comparison (Li et al., 2025); third, there is still a lack of comprehensive analysis on the relationship between exercise intervention parameters (such as exercise intensity, duration and intervention cycle) and the effect of depression improvement from the perspective of evidence-based medicine (Wang H. et al., 2025; Wang Y. Q. et al., 2025). For these reasons, a comprehensive evaluation of the literature is needed to determine the impact of exercise interventions on depressive levels in perimenopausal women.

Based on this, this study adopted the method of systematic review and Meta-analysis to comprehensively analyze the overall effect of exercise intervention on improving depressive symptoms in Chinese perimenopausal women, and further explore the differences in its impact from the aspects of exercise intensity, intervention cycle, exercise duration and frequency, putting forward the research hypothesis (H): exercise intervention can significantly improve the depressive level of Chinese perimenopausal women. This study contributes to the existing literature by focusing specifically on Chinese perimenopausal women, a population that has received relatively limited attention in previous evidence syntheses. In addition, subgroup analyses were conducted to explore whether exercise characteristics, including session duration, frequency, intensity, and intervention period, were associated with differences in intervention effects. These findings may provide useful evidence for the development of exercise-based strategies to improve depressive symptoms among perimenopausal women.

## 2 Research methods

### 2.1 Study framework

This study was reported in accordance with the PRISMA 2020 statement. The methodological framework was based on the Cochrane Handbook for Systematic Reviews of Interventions. The PICOS framework is shown in Table 1. Participants were Chinese women in the perimenopausal period with depressive symptoms. Studies were eligible if participants were identified as perimenopausal women according to the diagnostic criteria reported in the original studies. The review team relied on the classification provided by the original investigators and did not independently reassess menopausal status. The experimental intervention was any structured exercise therapy, defined as planned, repeated physical activity (e.g., aerobic exercise, mind–body exercise, dance, resistance training) with a minimum intervention duration of 4 weeks and at least 2 sessions per week. The control group received non-exercise interventions. The primary outcome was depressive symptoms measured by validated scales. Only randomized controlled trials (RCTs) were included.

**Table 1:** PICOS core question and inclusion criteria.

Core questions	Inclusion criteria
Study participants	Chinese Perimenopausal women with depression
Experimental intervention	Experimental group: structured exercise interventions, including aerobic exercise, walking, yoga, Tai Chi, Baduanjin, square dancing, and other planned physical activities, with clearly reported exercise protocols
Control intervention	Control group: non-exercise intervention
Outcome indicators	Depression-related indicators in perimenopausal women
Study design	Randomized controlled Trials (RCTs)

This systematic review was registered with the International Prospective Register of Systematic Reviews (PROSPERO, http://www.crd.york.ac.uk/PROSPERO) under registration number CRD420251271853.

### 2.2 Literature search strategy

A combination of subject headings and free words was used to search the Web of Science, PubMed, Cochrane Library, China National Knowledge Infrastructure (CNKI), Wanfang Database, and Chinese Science and Technology Periodicals Database (VIP), with the search period ranging from each database’s inception to December 31, 2025. The English search strategy was: (Perimenopausal women OR middle-aged women OR menopausal women) AND (exercise intervention OR physical exercise OR exercise OR physical activity) AND (depression OR mental health OR psychological health OR anxiety); the Chinese search strategy was: (围绝经期女性 OR 中年女性 OR 更年期女性) AND (运动干预 OR 体育锻炼 OR 运动 OR 体育活动) AND (抑郁 OR 身心健康 OR 心理健康 OR 焦虑情绪).

Inclusion criteria: ① Participants were Chinese perimenopausal women who met the diagnostic criteria for perimenopause and had confirmed depressive symptoms or a diagnosis of depression; ② Study design: randomized controlled trials (RCTs); ③ Interventions: the experimental group received exercise-based interventions, including but not limited to aerobic exercise, rope skipping, aerobics, Tai Chi, yoga, etc.; ④ Controls: the control group received routine care, health education, or no exercise intervention; ⑤ Outcome measures: at least one depression-related scale score (e.g., SCL-90, SDS, CES-D, etc.); ⑥ Publication type and language: publicly available Chinese or English literature; ⑦ Data integrity: complete data on exercise duration, frequency, and intervention period, with means, standard deviations, or equivalent statistical indicators available for meta-analysis.

Exclusion criteria: ① Participants who did not meet the diagnostic criteria for perimenopause or were not Chinese; ② Interventions focusing primarily on medication, psychotherapy, or other non-exercise approaches; ③ Outcome measures without depression-related scales; ④ Duplicate publications; only the version with the most complete data was retained.

### 2.3 Literature screening and data extraction

This study was conducted in strict accordance with the procedures specified in the Preferred Reporting Items for Systematic Reviews and Meta-Analyses (PRISMA, 2020) statement (Page et al., 2021). Literature screening, including title, abstract and full-text assessment, was conducted independently by two reviewers in duplicate. Data extraction was performed independently by two researchers using a uniformly designed form. The extracted information included first author, publication year, depression rating scale, basic characteristics of the participants (sample size, age), exercise intervention details (intensity, weekly frequency, duration per session, and intervention period), primary outcome measures, and relevant statistical data (mean and standard deviation). Studies without complete available data were excluded from the meta-analysis. After extraction, a third researcher checked the data for accuracy and consistency, and the final agreement rate for the selection and extraction results was 100%.

### 2.4 Literature quality assessment

Methodological quality assessment of the included literature was performed strictly using the Cochrane Risk of Bias tool for randomized controlled trials (RCTs) recommended in the Cochrane Handbook 5.1.0 (Higgins et al., 2011). The assessment covered six domains with a total of seven items: selection bias (including random sequence generation and allocation concealment), performance bias (blinding of investigators and participants), detection bias (blinded assessment of outcomes), attrition bias (completeness of outcome data), reporting bias (selective reporting of results), and other potential sources of bias. Each item was judged as “low risk of bias,” “high risk of bias,” or “unclear” according to the bias risk assessment criteria. Risk of bias assessment was completed independently by two reviewers. Any disagreements were resolved by a third researcher.

### 2.5 Statistical analysis

Review Manager 5.4 was used for risk of bias assessment, and Stata 16.0 was adopted to conduct the meta-analysis. The standardized mean difference (SMD) with 95% confidence interval (CI) was calculated as the pooled effect size. Heterogeneity across included studies was assessed using the I^2^ statistic and Chi-square (χ^2^) test, following the recommendations of the Cochrane Handbook for Systematic Reviews of Interventions. According to Cochrane guidance: I^2^ values of 0–40% may represent low heterogeneity, 30–60% may represent moderate heterogeneity, 50–90% may represent substantial heterogeneity, and 75–100% may represent considerable heterogeneity. Considering the clinical diversity (differences in exercise protocols, participant baseline characteristics) and methodological diversity across the included RCTs, a random-effects model was prioritized for all pooled analyses. A fixed-effects model would only be applied if both low statistical heterogeneity (I^2^ ≤ 40%) and consistent study design were observed.

Subgroup analyses were performed to explore sources of heterogeneity in the antidepressant effects of exercise among perimenopausal women, according to exercise duration, intensity, frequency, intervention period, depression assessment tool and exercise type. Notably, subgroup analyses were only conducted for categories containing 10 or more independent studies to ensure statistical reliability. Funnel plots were used to assess publication bias when ≥10 studies were included. Sensitivity analysis was conducted by omitting one study at a time to test the stability of the pooled results.

## 3 Results

### 3.1 Results of literature search

**Figure 1:**
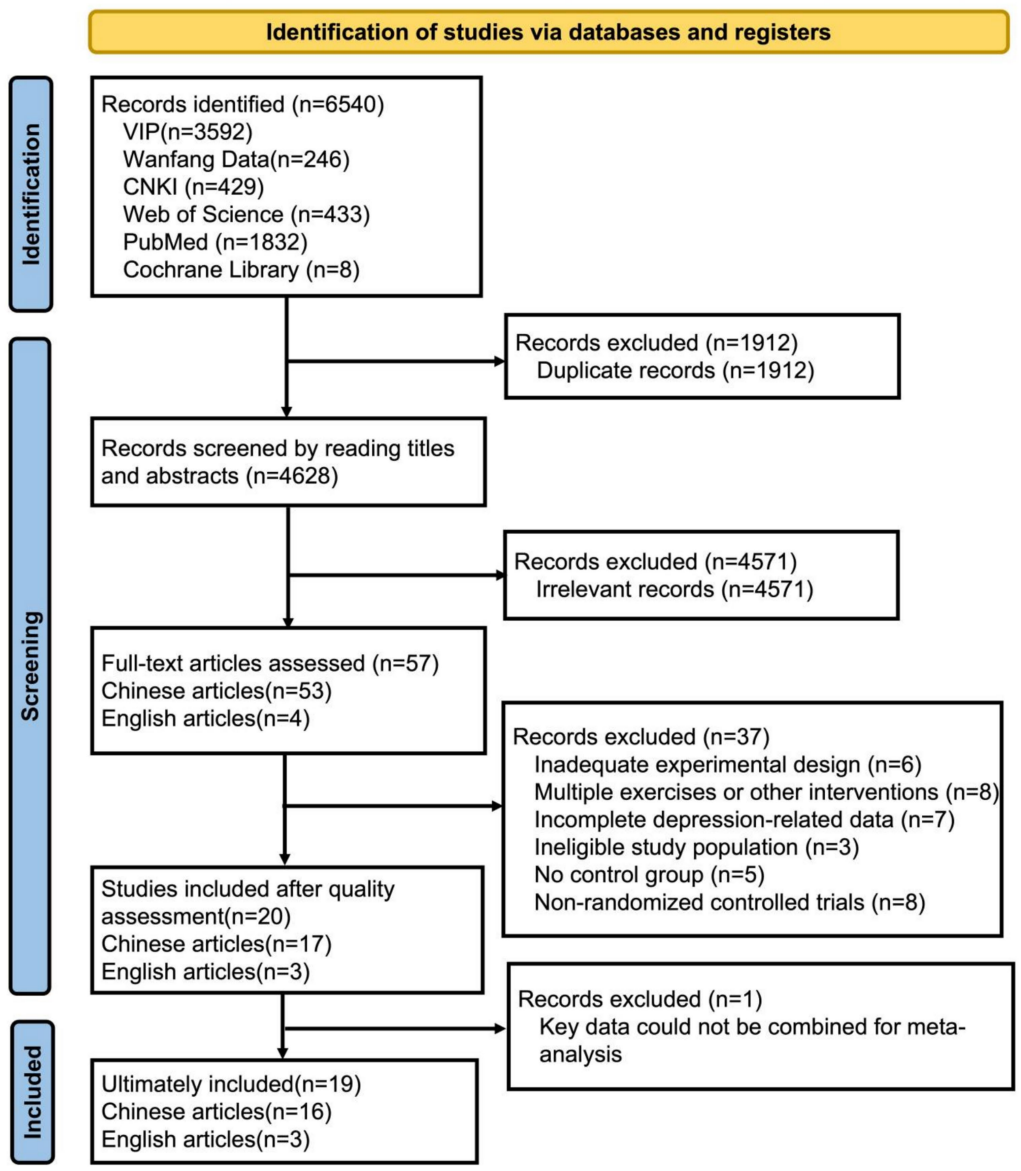
Flowchart of literature screening.

After retrieving records from six electronic databases, all citations were imported into EndNote software for deduplication, and 1912 duplicate records were removed. The remaining 4,628 unique records underwent independent dual screening of titles and abstracts by two reviewers. A total of 4,571 records were excluded at this stage due to irrelevant research themes inconsistent with our pre-defined PICOS inclusion criteria, leaving 57 articles eligible for full-text assessment. The complete screening workflow is illustrated in the PRISMA flow diagram following the PRISMA 2020 statement. A total of 19 studies were finally included, comprising 16 Chinese-language and 3 English-language studies. The literature screening process is illustrated in Figure 1.

### 3.2 Basic characteristics of included studies

A total of 19 studies involving 21 randomised controlled trials (RCTs) were included in this study, eighteen studies followed a standard two-arm design (one exercise group versus one control group), contributing one pairwise comparison each. The remaining study (Chen et al., 2017) was a three-exercise-arm RCT with one shared control group. To avoid duplicate counting of control participants when generating three separate pairwise comparisons, we evenly split the total sample size of the unified control group (*n* = 24) into three equal subsets of *n* = 8, with one subset matched to each individual exercise arm, with publication years ranging from 2011 to 2024. All participant ages ranged from 40 to 65 years, with the majority of studies reporting a mean age between 45 and 55 years. The interventions were all exercise-based, with diverse forms including aerobic exercises such as walking, rope skipping, and yoga (Guo and Chen, 2011; Ma et al., 2011; Zhang et al., 2014; Jia, 2016; Hu et al., 2017; Chen et al., 2017; Cao, 2020; Xu, 2022; Qin et al., 2024); dance-based exercises such as square dance and fitness dance (Guo and Zhang, 2013; Yi and Tan, 2013; Gao et al., 2016; Song, 2016; Li et al., 2017; Ding, 2018; Xu et al., 2019); resistance training (Chen et al., 2017; Zhang, 2021); and health qigong (Zheng and Chen, 2014; Shi et al., 2017; Chen et al., 2017); the control group received no exercise intervention or maintained their original lifestyle.

The sample size of each study ranged from 20 to 157 cases, and the sample size of a single group was 10 to 79 cases. The exercise intervention intensity was mainly low to moderate, classified according to original trial descriptions: low intensity was defined as 40–55% heart rate reserve (HRR) or Rating of Perceived Exertion (RPE) 9–11, and moderate intensity as 55–70% HRR or RPE 12–13. Each exercise session lasted 30 to 120 min, with an exercise frequency of 2 to 7 times per week, and an intervention period of 8 to 24 weeks. For the evaluation tools of depression level, 8 studies adopted SDS (Self-Rating Depression Scale), 6 studies used SCL-90 (Symptom Checklist 90), 4 studies employed KMI (Menopause Index Scale), 2 studies applied CES-D (Centre for Epidemiologic Studies Depression Scale), and 1 study used BDI (Beck Depression Inventory). All studies reported changes in depression levels before and after the intervention, which met the data requirements for meta-analysis. The specific basic characteristics of the included studies are shown in Table 2.

**Table 2:** Basic characteristics of included studies.

Study	Depression assessment scales	Exercise modalities	Experimental groups	Control al groups	Age	Exercise intensity (Low/Moderate)	Duration per session (min)	Frequency (sessions/week)	Intervention cycle (weeks)
Guo and Chen (2011)	CES-D	Rope Skipping	30	30	45–50	Moderate	45	3	12
Ma et al. (2011)	CES-D	Walking	46	50	45–55	Low	45	5	12
Guo and Zhang (2013)	SDS	Square Dance	29	29	45–55	Low	40	3–4	8
Yi and Tan (2013)	SDS	Fitness Dance	50	50	45–55	Moderate	40–60	5–7	24
Zheng and Chen (2014)	KMI	Taijiquan	40	40	40–55	Moderate	30–40	2	24
Zhang et al. (2014)	KMI	Walking	78	79	40–55	Low	30	3	12
Gao et al. (2016)	SDS	Square Dance	26	24	45–55	Moderate	60–90	5	12
Jia (2016)	SDS	Yoga	30	31	45–55	Moderate	90	3	24
Song (2016)	SCL-90	Aerobics	30	30	40–55	Moderate	45	3	12
Hu et al. (2017)	BDI	Walking	40	40	45–65	Low	60	3	16
Shi et al. (2017)	KMI	Taijiquan	40	40	45–55	Moderate	60–90	4–7	24
Chen et al. 1 (2017)	SDS	Jogging	25	8	45–55	Moderate	30–40	3–4	24
Chen et al. 2 (2017)	SDS	Resistance Training	24	8	45–55	Moderate	45	3–4	24
Chen et al. 3 (2017)	SDS	Qigong	27	8	45–55	Low	30	3–4	24
Li et al. (2017)	KMI	Square Dance	55	52	40–59	Low	60–120	5–7	12
Ding (2018)	SCL-90	Sports dance	30	30	45–55	Moderate	90	2	12
Xu et al. (2019)	SCL-90	Square Dance	39	40	45–55	Low	40	2	24
Cao (2020)	SCL-90	Yoga	20	20	45–59	Low	60	4	8
Zhang (2021)	SCL-90	Resistance Training	10	10	40–55	Low	60	3	12
Xu (2022)	SCL-90	Yoga	40	40	43–53	Moderate	60	3	12
Qin et al. (2024)	SDS	Yoga	38	38	44–60	Moderate	30	7	8

BDI, Beck Depression Inventory; CES-D, Center for Epidemiological Studies Depression Scale; SCL-90, Symptom Checklist-90; SDS, Self-rating depression scale; KMI, Kupperman Menopausal Index.

### 3.3 Quality assessment of included studies

**Figure 2:**
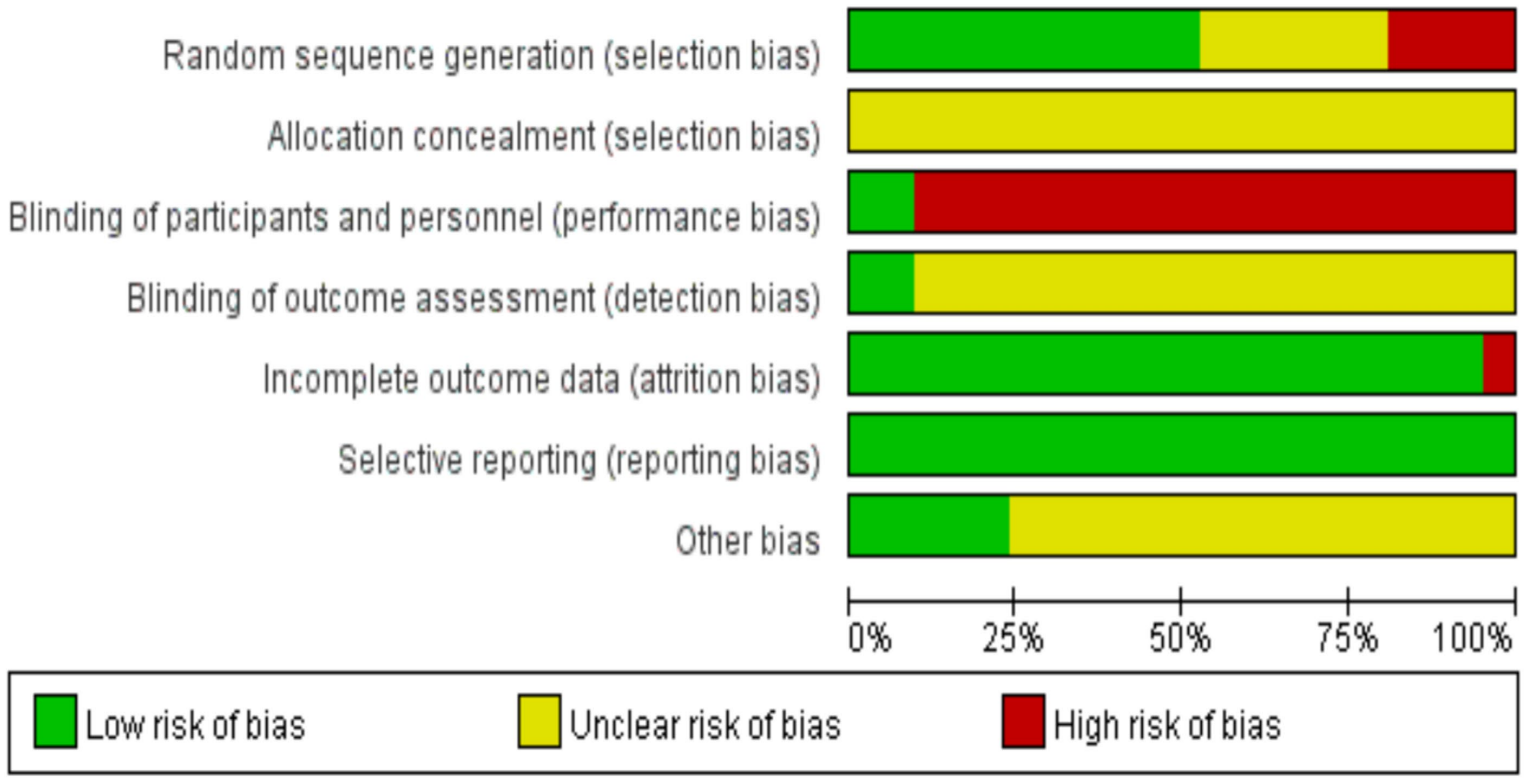
Proportion of risk of bias.

**Figure 3:**
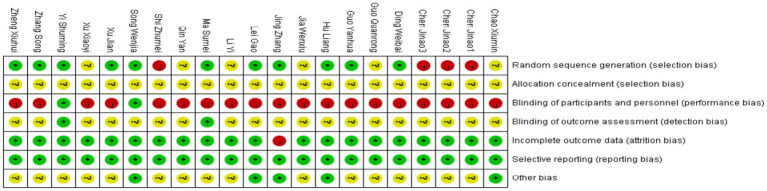
Results of risk of bias assessment for included studies.

Among the 19 RCTs included in this study, 5 were rated as low risk of bias (Ma et al., 2011; Yi and Tan, 2013; Gao et al., 2016; Song, 2016; Hu et al., 2017), and the rest were mainly rated as medium or high risk. This result is consistent with the general characteristics of RCTs on exercise intervention: ① Only one study implemented blinding for investigators, participants, and outcome assessors (Yi and Tan, 2013), Two studies achieved partial blinding one blinded participants (Song, 2016) and one blinded outcome assessors (Ma et al., 2011), However, complete blinding of participants or investigators is difficult in exercise intervention trials because participants can directly perceive the intervention, leading to a high risk of performance bias. ② Most studies did not describe the specific methods of allocation concealment in detail (e.g., opaque envelopes, central randomisation), and the risk of allocation concealment was mostly judged as “unclear.” ③ Although 11 studies mentioned “random allocation” (Ma et al., 2011; Guo and Zhang, 2013; Yi and Tan, 2013; Zheng and Chen, 2014; Zhang et al., 2014; Gao et al., 2016; Song, 2016; Hu et al., 2017; Ding, 2018; Xu et al., 2019; Zhang, 2021), some did not clearly specify the specific tools for random sequence generation (Guo and Zhang, 2013; Yi and Tan, 2013; Song, 2016; Hu et al., 2017) (e.g., random number table, computer-generated randomisation), resulting in inadequate reporting of randomisation methods. The above limitations are inherent characteristics of exercise intervention studies, rather than quality defects of the literature included in this study. The detailed distribution of the Cochrane risk-of-bias assessment is shown in Figures 2, 3. Subsequent sensitivity analysis showed that after excluding studies with low risk of bias, the direction and magnitude of the pooled effect size did not change significantly, suggesting that the core conclusion of this study (exercise improves physical and psychological symptoms in perimenopausal women) was not substantially altered by the risk of bias.

### 3.4 Meta-analysis

#### 3.4.1 Results of meta-analysis

This study included randomized controlled trials (RCTs) to systematically evaluate the effects of exercise interventions on depressive symptoms in perimenopausal women. Pre- and post-intervention depression data meeting eligibility criteria were extracted and analyzed via Stata 16.0. Given inconsistent depression assessment scales across trials, standardized mean difference (SMD) with 95% confidence interval (95% CI) was adopted as the pooled effect size. All pooled comparisons relied on post-intervention scores rather than change scores. This choice was justified by the fact that most trials fully reported post-intervention means and standard deviations, and baseline depression levels were balanced between groups at enrolment, making post-intervention outcomes appropriate for estimating net intervention effects.

**Figure 4:**
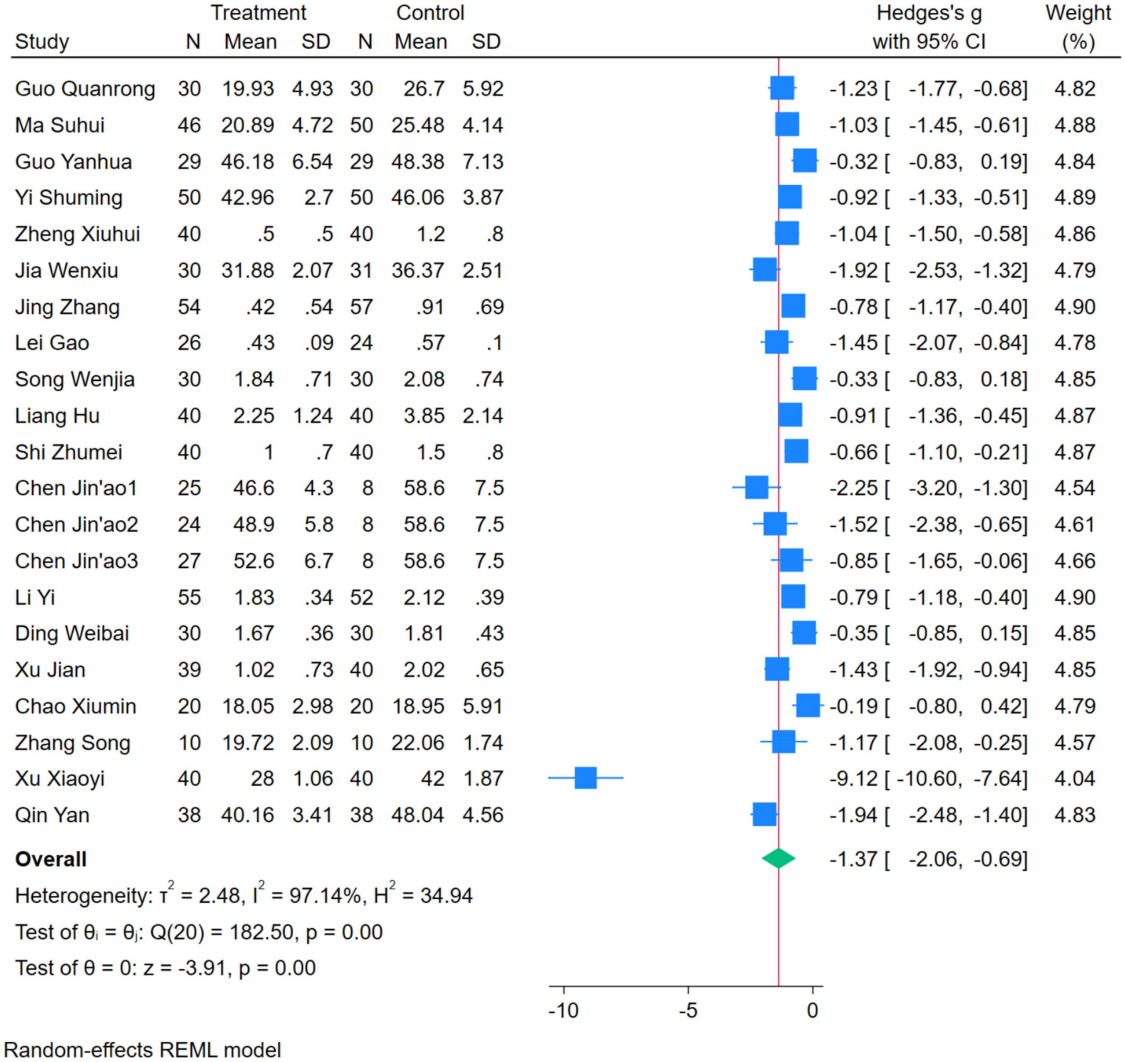
Results of meta-analysis.

A total of 21 RCTs with 1,398 participants were included in this meta-analysis to evaluate the effect of exercise intervention on depression in perimenopausal women. As shown in Figure 4, forest plot results indicated high heterogeneity across studies (I^2^ = 97.14%, *p* < 0.001); therefore, the random-effects model was adopted for pooled analysis. The statistical results showed that the pooled effect size was SMD = −1.37 (95% CI: −2.06 to −0.69), *p* < 0.001, indicating a statistically significant difference. These findings suggested that exercise could significantly reduce the severity of depression in perimenopausal women.

#### 3.4.2 Publication bias analysis

**Figure 5:**
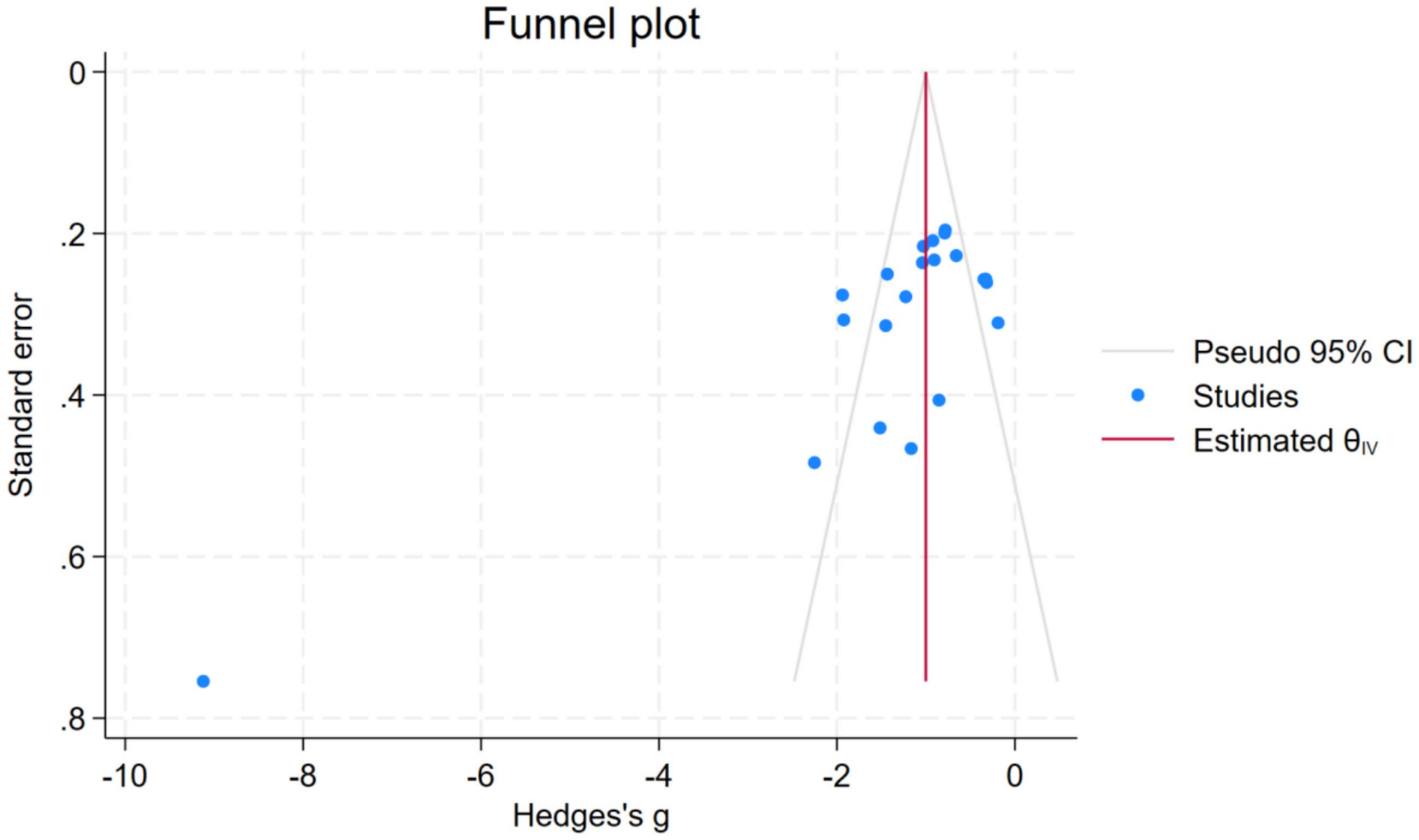
Funnel Plot of depression severity distribution.

The results of the publication bias analysis for depressive symptoms in perimenopausal women are presented in Figure 5. Although the funnel plot of the 21 included studies was not perfectly symmetrical, most studies were distributed around the pooled effect size, with slight asymmetry observed among several small-sample studies. This asymmetry may be attributable to both potential publication bias and substantial between-study heterogeneity. Given the considerable variation in exercise characteristics and study designs across the included studies, heterogeneity may have contributed to the observed funnel plot asymmetry.

We performed Begg’s test and Egger’s test to assess potential publication bias. The results of Begg’s test showed a *p*-value = 0.028, and the adjusted p-value after continuity correction was 0.028, both of which were less than 0.05, indicating statistically significant publication bias. The results of Egger’s test showed that the p-value for the intercept coefficient was 0.001 (95% CI), also indicating the presence of certain publication bias. While the trim-and-fill method imputed missing studies and produced an adjusted effect size that remained statistically significant, this analysis should be interpreted with caution due to the presence of substantial heterogeneity. Therefore, publication bias cannot be completely ruled out.

#### 3.4.3 Subgroup analysis based on exercise characteristics

Subgroup analyses stratified by single-session duration, weekly frequency, exercise intensity and total intervention cycle were conducted to explore potential sources of between-study heterogeneity. Full results are summarized in Table 3. All individual subgroups yielded statistically significant within-group pooled effects (see two-tailed *p* values in Table 3). Formal tests for overall differences between subgroups were further calculated for each moderator:

Single-session exercise duration: *p* = 0.551.
Weekly exercise frequency: *p* = 0.493.
Exercise intensity: *p* = 0.142.
Total intervention cycle: *p* = 0.687.

**Table 3:** Subgroup analyses of exercise duration, exercise frequency, exercise intensity, and exercise cycle.

Subgroup category	Homogeneity test			Subgroup level	No. of studies	SMD (95% CI)	Two-tailed test		Tests for subgroup differences
	X^2^	*P*	*I* ^2^				*Z*	*P*	*p*
Exercise duration per session (min)	28.71	0.000	81.27%	≤40	7	−1.19 [−1.66, −0.71]	4.89	0.000	0.551
	8.64	0.07	54.6%	40 < X < 60	5	−0.95 [−1.29, −0.61]	5.42	0.000	
	143.90	0.000	98.84%	≥60	9	−1.79 [−3.52, −0.06]	2.02	0.000	
Exercise frequency (sessions/week)	165.32	0.000	97.82%	≤4	15	−1.50 [−2.51, −0.49]	2.92	0.000	0.493
	16.99	0.000	73.49%	5 ≥ X≧7	6	−1.10 [−1.47, −0.73]	5.90	0.000	
Exercise intensity (low/moderate)	15.52	0.05	50.4%	Low	9	−0.83 [−1.07, −0.58]	6.69	0.000	0.142
	159.12	0.000	98.27%	Moderate	12	−1.83 [−3.08, −0.59]	2.9	0.000	
Total intervention cycle (weeks)	158.50	0.000	98.59%	≤12	12	−1.50 [−2.79, −0.22]	2.29	0.02	0.687
	21.12	0.01	64.32%	>12	9	−1.20 [−1.52, −0.89]	7.55	0.000	

The *P* value under Two-tailed Test represents statistical significance for pooled effect within each individual subgroup. The column “Test for subgroup difference (P)” reports the overall p value testing whether effect magnitudes differ significantly across subgroups within one moderator category.

All four tests for subgroup differences returned *p* > 0.05, indicating no statistically significant disparities in pooled effect sizes across respective subgroups. Substantial residual heterogeneity remained within nearly all subgroups (*I^2^* range: 50.4–98.84%), demonstrating these four moderators could not fully explain overall study heterogeneity. Several subgroups contained fewer than 10 comparison pairs, so these stratified results are interpreted cautiously due to limited statistical power. In addition, statistical significance of within-subgroup effect sizes only indicates exercise outperformed control conditions, and cannot be equated to superior clinical effectiveness across different exercise regimens. All subgroup patterns are regarded as exploratory observations only, and no definitive exercise prescription recommendations are proposed herein.

### 3.5 Sensitivity analysis

A leave-one-out sensitivity analysis was performed by omitting one study at a time and recalculating the pooled effect size. The results showed that after omitting any single study, the pooled effect sizes ranged from −1.44 to −1.01, and all 95% confidence intervals did not cross the null line (SMD = 0). The direction of the effect sizes was completely consistent with the original analysis (all negative, indicating that exercise alleviated depression). These findings indicate that the pooled results of this study are robust and not excessively influenced by any single study.

## 4 Discussion

Through a meta-analysis of 21 RCTs, this study confirmed that exercise intervention significantly reduced depression levels in perimenopausal women (SMD = −1.37, 95% CI: −2.06 to −0.69, *p* < 0.001). This finding is consistent with the core results of similar studies worldwide (Xu et al., 2025; Dong and Hwang, 2025), further confirming the effectiveness of exercise as a non-pharmacological intervention to improve emotional symptoms during the perimenopausal period. It also provides new evidence-based support for the non-pharmacological management of emotional disorders in perimenopausal women.

Subgroup analyses revealed that intervention programs with different exercise duration, frequency, intensity, and period all produced significant antidepressant effects. Among them, moderate-intensity exercise and sessions lasting ≥60 min yielded the largest effect sizes. In contrast, programs featuring low intensity, 5–7 sessions per week, and an intervention period of >12 weeks showed greater stability of effects. These observations must be interpreted with great caution. High residual heterogeneity remained across all subgroups. As statistical differences do not equal superior clinical efficacy, we do not propose targeted exercise recommendations for clinical practice. The trends observed in this work are only exploratory, which are partially in line with related research conclusions (Dong and Lee, 2023; Yue et al., 2025; Zeng et al., 2023).

The RCTs included in this study showed high heterogeneity (I^2^ = 97.14%). Although varied depression assessment tools may contribute to cross-study differences, our predefined subgroup factors failed to fully resolve the high heterogeneity. Given that residual heterogeneity persisted after stratification, we cannot confirm the exact sources of between-study variation, even though prior studies have discussed relevant influencing factors (Posadzki et al., 2020). Sensitivity analysis indicated the overall pooled result was relatively stable. Funnel tests suggested potential publication bias, which may also affect the reliability of the findings.

Limitations of this study include certain deficiencies in the methodological quality of the included RCTs. Most studies did not describe allocation concealment methods in detail, and complete blinding was difficult to implement in exercise interventions. These are not defects of the present study but inherent methodological challenges in RCTs of exercise interventions. In addition, this study did not include studies combining exercise with other interventions. Existing evidence has shown that exercise combined with psychological intervention can further improve depressive symptoms in perimenopausal women (Wang H. et al., 2025; Wang Y. Q. et al., 2025), which provides a direction for future extensions of this study. Future studies should further optimise trial design, such as adopting central randomization systems to standardise allocation concealment and developing standardized exercise intervention protocols. Meanwhile, the synergistic effects of combined interventions, such as exercise plus cognitive behavioral therapy or traditional Chinese medicine conditioning, can be explored to more precisely alleviate depressive symptoms in perimenopausal women.

## 5 Conclusion

In summary, this meta-analysis of 21 RCTs revealed that exercise interventions are statistically associated with reduced depressive symptoms among perimenopausal women, and exploratory subgroup trends were observed across different exercise parameters. Due to the substantial overall and within-subgroup heterogeneity, as well as existing methodological limitations of the included studies, these findings can only serve as preliminary evidence for clinical reference rather than definitive guidance. We suggest that practitioners take individual physical tolerance, exercise preferences and health status into full consideration when designing personalized exercise plans. Combining moderate- and low-intensity exercise with regular, long-term activity may help relieve depressive symptoms. Targeted guidance and community support can also help improve exercise adherence.

## Data Availability

The datasets presented in this study can be found in online repositories. The names of the repository/repositories and accession number (s) can be found in the article/supplementary material.
